# Application and assessment of grouted-in pressure transducers to determine long-term in situ hydrogeologic properties of argillaceous aquitards

**DOI:** 10.1007/s10040-026-03091-6

**Published:** 2026-05-27

**Authors:** Laura A. Smith

**Affiliations:** Department of Civil, Geological, and Environmental Engineering, University of Saskatchewan, Saskatoon, SK Canada

**Keywords:** Aquitard, Fully grouted method, Groundwater monitoring, Hydraulic properties, Numerical modeling

## Abstract

Characterizing the in situ hydrogeologic properties of aquitards is difficult due to their low hydraulic conductivity (*K*) and slow response times. Consequently, numerical models often rely on laboratory determined values for geotechnical and hydrogeological parameters, which may be unreliable due to sample disturbance. This study shows that fully grouted, vibrating wire pressure transducers (VWPs) can be used to estimate the in situ compressibility (*α*), specific storage (*S*_S_), and in situ horizontal hydraulic conductivity (*K*_h_) of overconsolidated aquitards by analyzing pore pressure responses to barometric pressure fluctuations and post-installation recovery. While these methods have been used in shallow or short-term studies, their application in deeper, low-*K* formations with long-term monitoring remains limited. This study presents results from 34 VWPs installed across five boreholes at a single site (site 2) and compares them with previously published data from a similar site (site 1) in the Williston Basin (WB) where the methods were first developed and tested. The dataset provides a rare, high-resolution record of pore pressure behavior in glacial till and Cretaceous shale over monitoring periods of up to 16 years. The findings demonstrate the reproducibility of the methods across different lithologies and support their use in estimating representative hydraulic parameters for regional-scale models. This work adds to the growing body of research supporting in situ methods for aquitard characterization and highlights the value of long-term monitoring in improving the understanding of low-*K* systems in sedimentary basins.

## Résumé

Caractériser les propriétés hydrogéologiques in situ des aquitards argileux est difficile en raison d’une conductivité hydraulique (*K*) faible et de temps de réponse longs. En conséquence, les modèles numériques reposent souvent sur des valeurs déterminées en laboratoire en ce qui concerne les paramètres géotechniques et hydrogéologiques, qui peuvent ne pas être fiables du fait de l’altération de l’échantillon. L’étude montre que les transducteurs de pression à corde vibrante (TPCV) peuvent être utilisés pour estimer la compressibilité in situ (α), l’emmagasinement spécifique (*S*_*s*_) et la conductivité hydraulique horizontale in situ (*K*_h_) des aquitards sur consolidés, par l’analyse des réponses de la pression interstitielle aux fluctuations de la pression barométrique et au rétablissement après installation. Alors que ces méthodes ont été utilisées dans des études de subsurface ou de court terme, leur application à des formations plus profondes et de faible *K, *avec un suivi sur le long terme, reste limitée. L’étude présente les mesures de 34 TPCV mis en place dans cinq forages, dans un site unique (Site 2) et les compare aux données publiées précédemment sur un site similaire ( Site 1) du Bassin de Williston (BW) où les méthodes ont été antérieurement développées et testées. La base de données fournit une relation précieuse et de haute résolution des réponses de la pression interstitielle dans une moraine glaciaire et un schiste crétacé pendant des périodes de surveillance allant jusqu’à 16 ans. Les résultats démontrent l’applicabilité des méthodes aux différentes lithologies et encourage leur utilisation pour l’estimation des paramètres hydrauliques représentatifs dans des modèles à l’échelle régionale. Ce travail s’ajoute au corpus croissant de la recherche qui appuie les méthodes in situ pour la caractérisation des aquitards et met en lumière l’intérêt de la surveillance sur le long terme pour l’amélioration de la compréhension des systèmes de faible *K* dans les bassins sédimentaires.

## Resumen

La caracterización de las propiedades hidrogeológicas in situ de los acuitardos es difícil debido a su baja conductividad hidráulica (K) y a los lentos tiempos de respuesta. En consecuencia, los modelos numéricos suelen depender de valores determinados en laboratorio para parámetros geotécnicos e hidrogeológicos, que pueden ser poco fiables debido a la perturbación de la muestra. Este estudio muestra que los transductores de presión de hilo vibrante (VWP) completamente sellados pueden usarse para estimar la compresibilidad in situ (*α*), el almacenamiento específico (*SS*) y la conductividad hidráulica horizontal in situ (*Kh*) de los acuítardos sobreconsolidados, analizando las respuestas de presión de los poros a fluctuaciones de presión barométrica y la recuperación posterior a la instalación. Aunque estos métodos se han utilizado en estudios superficiales o a corto plazo, su aplicación en formaciones más profundas y con bajo* valor de K*, con monitoreo a largo plazo, sigue siendo limitada. Este estudio presenta los resultados de 34 VWP instalados en cinco pozos en un solo sitio (Sitio 2) y los compara con datos previamente publicados de un sitio similar (Sitio 1) en la Cuenca de Williston (WB), donde se desarrollaron y probaron por primera vez los métodos. El conjunto de datos proporciona un registro singular y de alta resolución del comportamiento de la presión de poros en till glaciar y lutita cretácica durante periodos de seguimiento de hasta 16 años. Los hallazgos demuestran la reproducibilidad de los métodos a través de diferentes litologías y respaldan su uso en la estimación de parámetros hidráulicos representativos para modelos a escala regional. Este trabajo contribuye al creciente cuerpo de investigación que apoya métodos in situ para la caracterización de los acuitardos y destaca el valor del monitoreo a largo plazo para mejorar la comprensión de los sistemas bajos* en K* de cuencas sedimentarias.

## Abstract (zh)

由于弱透水层的渗透系数（*K*）较低、响应时间较长，其原位水文地质参数表征具有较大难度。因此，数值模型通常依赖室内试验测定的岩土工程和水文地质参数，而这些参数可能因取样扰动而存在较大不确定性。本研究表明，通过分析孔隙压力对气压波动的响应以及传感器安装后的压力恢复过程，全灌浆埋设的振弦式压力传感器（vibrating wire pressure transducers，VWPs）可用于估算超固结弱透水层的原位压缩系数（*α*）、贮水率（*S*_S_）和原位水平渗透系数（*K*_h_）。尽管上述方法已在浅部或短期监测研究中得到应用，但其在更深部、低渗透性地层中的长期监测应用仍较为有限。本研究给出了单一研究场地（场地2）内5个钻孔中安装的34个振弦式压力传感器的监测结果，并将其与Williston盆地（Williston Basin，WB）内一个相似场地（场地1）已发表的数据进行对比；相关方法最早即在场地1得到建立和验证。该数据集提供了冰碛黏土和白垩纪页岩中孔隙压力行为的少见高分辨率记录，监测时长最长达16年。研究结果表明，该方法在不同岩性条件下具有良好的可重复性，并支持其用于估算区域尺度模型所需的代表性水力参数。本研究进一步丰富了利用原位方法表征弱透水层特征的相关研究成果，并强调了长期监测对于加深认识沉积盆地低渗透性系统的重要价值。

## Resumo

Caracterizar as propriedades hidrogeológicas in situ dos aquítardos é difícil devido à sua baixa condutividade hidráulica (*K*) e tempos de resposta lentos. Consequentemente, modelos numéricos frequentemente dependem de valores determinados em laboratório para parâmetros geotécnicos e hidrogeológicos, que podem ser pouco confiáveis devido à perturbação da amostra. Este estudo mostra que transdutores de pressão de fio vibratório (TPFVs) totalmente rejuntados podem ser usados para estimar a compressibilidade in situ (*α*), armazenamento específico (*S*_*S*_*)* e condutividade hidráulica horizontal in situ (*K*_*h*_) de aquítardes superconsolidados, analisando respostas de pressão nos poros a flutuações de pressão barométrica e recuperação pós-instalação. Embora esses métodos tenham sido usados em estudos rasos ou de curto prazo, sua aplicação em formações mais profundas e com baixo* índice de K*, com monitoramento de longo prazo, permanece limitada. Este estudo apresenta resultados de 34 TPFVs instalados em cinco furos em um único local (Site 2) e os compara com dados publicados anteriormente de um local semelhante (Site 1) na Bacia Williston (BW), onde os métodos foram inicialmente desenvolvidos e testados. O conjunto de dados fornece um registro raro e de alta resolução do comportamento da pressão dos poros em tilito glacial e xisto do Cretáceo durante períodos de monitoramento de até 16 anos. As descobertas demonstram a reprodutibilidade dos métodos em diferentes litologias e apoiam seu uso na estimativa de parâmetros hidráulicos representativos para modelos em escala regional. Esse trabalho contribui para o crescente corpo de pesquisas que apoiam métodos in situ para caracterização de aquitardos e destaca o valor do monitoramento de longo prazo para melhorar a compreensão de sistemas de baixo índice de* K*.

## Introduction

Groundwater flow and contaminant transport through high hydraulic conductivity (*K* ≥ 10^–8^ ms^−1^) units, or aquifers, in sedimentary basins are characterized using a combination of hydraulic and geochemical analyses. However, numerous studies (Corbet and Bethke 1992; Neuzil 1986; Smith et al. 2013, 2016; van der Kamp 2001) have highlighted the challenges associated with estimating in situ hydrogeologic parameters of low-*K* argillaceous (clay-rich) formations, commonly referred to as aquitards. Conventional field-based methods, such as pump or slug tests, are often impractical in aquitards with low-*K* due to response times that can span from years to decades. Moreover, conventional laboratory testing of low-*K* samples tend to overestimate one-dimensional (1-D) constrained compressibility (*m*_v_), bulk compressibility (*α*), and specific storage (*S*_S_), while underestimating *K*, often by orders of magnitude (Smith et al. 2013, 2016; van der Kamp and Schmidt 1997). Characterization of the hydrogeologic properties of aquitards is foundational to our understanding of regional groundwater flow systems. Aquitards act as confining units by protecting fresh water sources from contaminants in adjacent aquifers and serve as caprock formations to trap hydrocarbons and prevent them from migrating upward in many sedimentary basins. In addition, thick basinal aquitards are considered attractive host formations for the potential sequestration of hazardous wastes (Hendry et al. 2015; Mazurek et al. 2011; Neuzil 2013).

High-resolution pore pressure data can be obtained from vibrating wire pressure transducers (VWPs) installed in aquitards using the fully grouted method (FGM) and can be used to determine hydrogeologic properties (*m*_v_, *α*, *K*, and *S*_S_) of argillaceous aquitard formations (Smerdon et al. 2014; Smith et al. 2016, 2013). Data obtained from fully grouted VWPs are being increasingly applied across many hydrogeologic conditions, ranging from characterizing multi-layered systems (Marefat et al. 2015; Zhuang et al. 2017, 2015), tracking changes in specific storage of overburden rock during mining (David et al. 2017), determining stiffness degradation in shale during slow-moving landslides (Antunes 2020), delineating fractured sedimentary rocks (Meyer 2013), determining the effect of seismic activity on low permeability formations (Kelly et al. 2018), and used to detect hydraulic responses indicative of fractures in clay (Young et al. 2022). However, there is limited published information regarding the long-term (> 5 years) performance of these installations, the potential installation-related errors, or the extent to which in situ analysis may vary over time. Moreover, owing to the relatively recent development of these in situ techniques, hydrogeologic parameters for aquitards used in numerical modeling and subsequent interpretations of groundwater flow or contaminant transport are still predominantly derived from traditional laboratory tests conducted on disturbed or damaged core samples.

This paper aims to refine the estimated range of in situ hydraulic properties (e.g., *K*, *α*, *S*_S_) characteristic of argillaceous aquitards (e.g., glacial till and shale) by developing a database for analogous formations worldwide without relying on data obtained from core samples. Specifically, the study objectives are to:

1. Present and evaluate the use of in situ methods for determining hydraulic properties using 34 additional VWPs installed in glacial till and Pierre Shale in the WB (site 2, 200 m depth) and compare these findings with long-term data obtained from 10 previously published VWPs (site 1, 325 m depth), as well as with values reported in the literature. This comparison aims to assess the effectiveness of the in situ monitoring methods developed at site 1 and to contextualize the findings within the broader range of values reported in the literature for similar geomaterials using field tests, laboratory measurements, or numerical modeling;
2. Assess the long-term stability and reliability of the grouted-in VWPs used for pore pressure monitoring and analyze how pore pressure varies with depth over time in the same borehole; and,
3. Evaluate the effectiveness of these methods in glacial till, shale, and fractured shale by characterizing pore pressure responses unique to each formation type.

## Background

### The fully grouted method (FGM)

Traditional piezometer installations typically involve backfilling the borehole with various materials (e.g., a sandpack around the piezometer tip and a bentonite seal above and below). The fully grouted method (FGM) seals the entire borehole with a cement-bentonite grout mixture, including the area around the instrument. VWPs are attached to a pipe at predetermined depths at surface, then lowered into the borehole. Grout is then pumped down the pipe and fills the borehole from the bottom to ground surface (i.e., bottom upward), removing the need for various types of geomedia and volume/depth calculations to ensure the piezometer tip is encased in a high-*K* material.

The development of diaphragm-type pressure transducers with the ability to measure small fluid volume changes over short distances enables the VWPs to respond quickly to changes in formation pore pressure despite the grout annulus. The advantages of using fully grouted VWPs in place of traditional piezometers include their ease of installation, applicability to low-*K* formations, and ability to install multiple VWPs in a single borehole to provide high-resolution vertical data profiles. Further, there may be an argument supporting grouted in piezometers to be classified as abandoned borings and remove the responsibility of long-term maintenance of the well. While the FGM has many benefits, it is extremely important to achieve the target grout *K* by mixing precise proportions of water, bentonite, and cement. Improper grout mixing can lead to water bypassing through the annular space (i.e., short circuiting), potentially compromising the borehole’s integrity and making it unusable (Contreras et al. 2007, 2012; McKenna 1995; Mikkelsen and Green 2003; Mikkelsen 2002; Vaughan 1969). A more recent study by Young et al. (2022) investigated this further by installing several multilevel VWP stacks with different backfills (including a fully grouted cement bentonite grout) and their influence on pore pressure measurements, highlighting the importance of appropriate installation materials for specific circumstances.

The literature supports the FGM for VWP installations in steady-state conditions with the caveat that the *K* of the grout must be within 2–3 orders of magnitude of the host formation (Contreras et al. 2007, 2012; McKenna 1995; Mikkelsen and Green 2003; Mikkelsen 2002; Vaughan 1969). When the grout’s *K* falls outside the target range, the likelihood of hydraulic ‘short-circuiting’ through the grout annulus increases. It is also important to note that a stiffness contrast between the grout and the surrounding formation may influence the short-term pore pressure response following installation, particularly if the grout is less stiff and deforms more readily under barometric loading. However, this effect is limited to the early post-installation period during grout curing and pore pressure recovery. If grout stiffness were significantly lower than expected or if grout quality were inconsistent, some uncertainty could be introduced into the early response; however, this limitation is minimized if the loading efficiency (*γ*) is evaluated only after long-term stabilization.

To verify that the grout is within the targeted *K* range, the stacked VWPs should exhibit distinct pore pressure responses to known barometric pressure fluctuations. If the pore pressure responses are identical, or nearly so, it is indicative of short-circuiting along the borehole annulus (Smith et al. 2013). The reliability of the FGM under transient conditions (e.g., embankment construction, complex stratigraphic sequences, and seepage areas) is increasingly viewed as not reliably applicable and remains under considerable scrutiny. Recent work by Marefat et al. (2014, 2015, 2019) examined FGM performance under a range of hydraulic conditions, focusing on the influence of grout to soil permeability and vertical hydraulic gradients (along with the geometry of the borehole). Their findings indicate that, in most field settings where the vertical gradient is less than 1, the grout permeability may exceed that of the surrounding formation by up to an order of magnitude with minimal error. A recent synthesis and finite-element assessment demonstrated that the FGM can yield considerable measurement errors under rapid transient pore pressure changes and provided practical criteria for when FGM may not be appropriate (Martens 2025). Further examination suggests that while a grout-to-formation permeability ratio of approximately 100 may be acceptable for steady-state conditions, more complex installations where transient flow is a concern require a permeability ratio closer to 10 to reduce the risk of hydraulic short circuiting (Martens 2025).

It is also important to note that errors are likely to be higher near stratigraphic interfaces (Marefat et al. 2015; Martens et al. 2020). For the purposes of this study, the VWPs installed using the FGM are in relatively stable conditions and therefore cannot provide clarity on the applicability of the FGM in transient, higher *K* conditions.

## Geologic setting

**Fig. 1:**
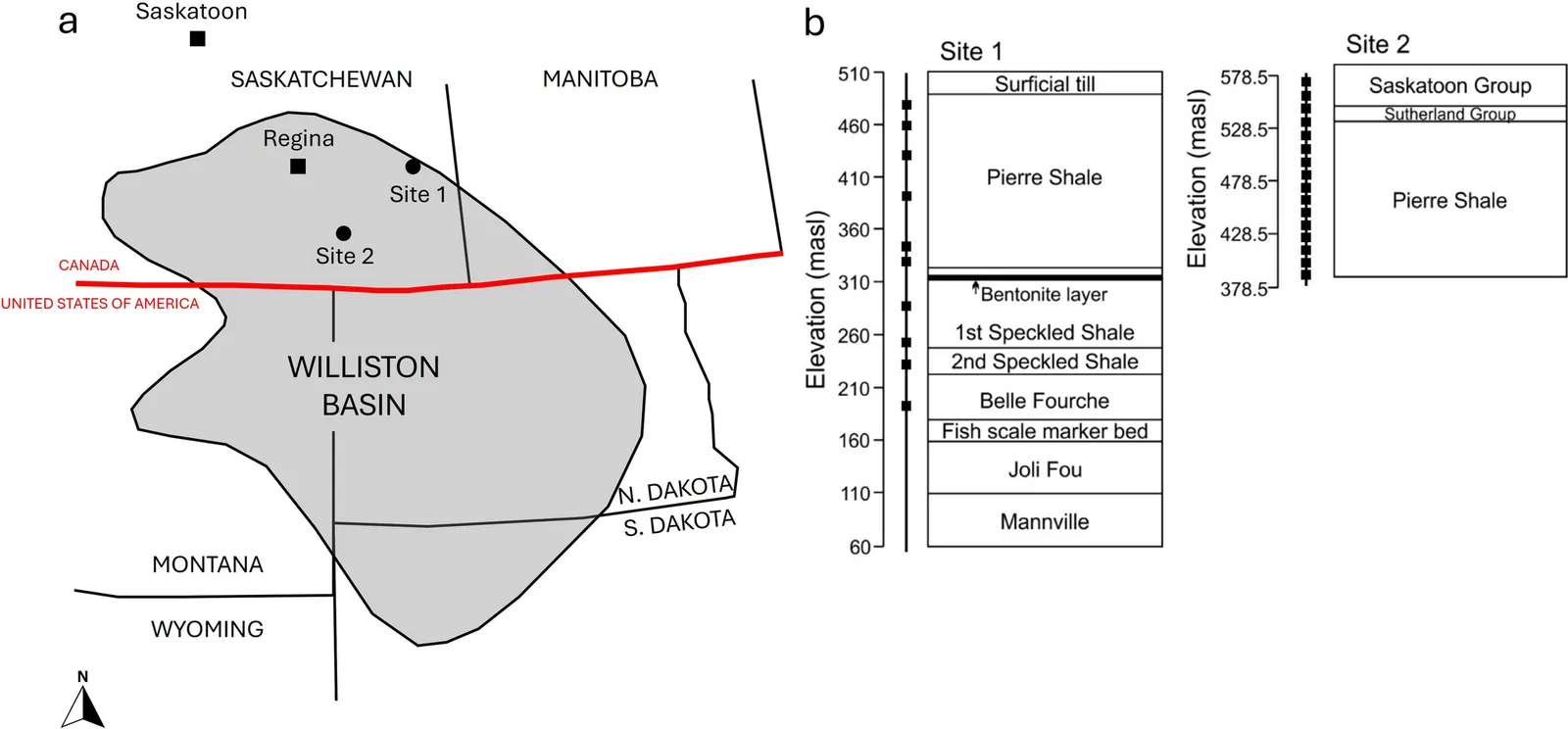
Map of the Williston Basin approximate boundaries through Canada and the United States (**a**). The locations of site 1 and site 2 are shown on the map, and the corresponding stratigraphic sections of each borehole are illustrated with transducer locations identified by a black square (■) (not to scale) (**b**). Only one location from site 2 is presented here and although the till/shale contact varies from 35–70 m BG at site 2, this profile is intended to illustrate the high frequency of VWP installations through both the till and the Pierre Shale (at 10 m intervals)

The Williston Basin (WB) is a sedimentary basin in North America that spans roughly 250,000 km^2^ of parts of North Dakota, South Dakota, Montana, Manitoba, and Saskatchewan (Vigrass 2006) (Fig. 1a). The WB extends approximately 764 km from north–south and 480 km from east–west, with the majority of its area located in the United States and centered near the city of Williston, North Dakota. The basin consists of a sequence of sedimentary material (e.g., limestone, sandstone, siltstone, shales, and evaporites) deposited since the Cambrian and is more than 4500 m thick in some areas (Gerhard et al. 1982). The shallowest formations in the WB, and the primary focus of this study, consist predominantly of nonmarine Quaternary aged glacial till, which is underlain by thick sequences of Cretaceous aged marine deposits (shale) (Sandberg 1962). The regionally extensive Mannville Group represents the oldest Cretaceous deposits in the WB and typically consists of alternating sand/shale in most areas of the Canadian portion.

### Study sites

The two sites examined in this study are separated by approximately 350 km within the WB. Site 1 is located on the northeast edge of the WB where the surficial till is thin (< 10 m) compared to regions closer to the center of the basin (Fig. 1a). The expected thickness of the Pierre Shale at site 1 is also thinner on the edge of the basin so it was economically possible to drill into the underlying formations (including the 1 st and 2nd Speckled Shale, and Belle Fourche Formations) where VWPs could also be installed. The elevation of ground surface at site 1 is 510.3 m above sea level. Site 2 is located approximately 350 km southwest of site 1 and closer to the center of the Canadian portion of the WB (Fig. 1a), where both the surficial till (54 m) and the underlying Cretaceous shale (940 m) are thicker than site 1 (Fig. 1b). Five boreholes between 100 and 200 m depth were completed within an 18 km^2^ area at site 2. The thickness of the overlying till (Saskatoon and Sutherland Group) at site 2 allowed for the installation of multiple VWPs in the till as well as the top 150 m of the Pierre Shale. The elevation of ground surface in the region of site 2 range from 569 to 580 m above sea level.

#### Background and reference data

Several manuscripts have been published focusing on various aspects of site 1 and site 2 (Hendry et al. 2013; Smith et al. 2013, 2016, 2018). All relevant studies are referenced in the discussion. The results from site 2 are compared to those from site 1 where appropriate, with clear citation of prior work. Although core samples from both sites were collected (including 1-D consolidation and triaxial permeability testing), those results have not been reproduced here as they are reported in Smith et al. (2013, 2016, 2018). These studies are cited and discussed in the relevant sections. The only previously published data from site 2 included in this paper is the *α* profile, which was originally presented in Smith et al. (2018) in the context of stress behavior observed in laboratory tests and its implications for in situ conditions.

## Materials and methods

### Drilling and installation of transducers

In 2009 a single borehole was continuously cored at site 1 using a Failing CF-15 Top Drive rotary drill rig to a total depth of 325 m below ground (m BG). Ten Geokon 4500S and 4500SH stainless steel VWPs were attached to a 51 mm steel tremie pipe at depths of 25, 50, 75, 125, 175, 185, 225, 255, 275, and 325 m BG. Effort was made to prevent cavities or areas between the wires by tightly securing them to the tremie pipe individually. Consistent with the FGM, a bentonite/cement grout mixture was pumped down the tremie pipe until grout returned to surface, indicating the entire borehole was filled with grout and the transducers were secured in place. The grout was a mixture of 4% bentonite-96% cement and was tested regularly during installation to ensure the specific gravity was approximately 1.7 (density of 1700 kg/m^3^), corresponding to a water to cement ratio of about 0.68:1 by mass. This ratio differs from the commonly referenced 2.5:1 ratio for ‘hard soils’ (Mikkelsen and Green, 2007; ISO, 2020) because this ratio would produce grout with a *K* near 10^–8^ m/s, which is much higher than the host formation’s expected *K* of < 10^–11^ m/s. For this reason, a lower water to cement ratio of 0.68:1 was used to ensure the permeability of the grout was within an acceptable range of the adjacent formation. Although ISO 18674-4 (2020) now lists typical grout mixes for FGM piezometers, these installations (2009–2015) predate the standard; moreover, the recipe for hard soils would yield grout *K* values (~ 10^–8^ m/s) that are several orders higher than the aquitard *K* targeted here (< 10^–11^). Samples of the grout were collected at depths corresponding to approximately 46, 182, and 317 ± 10 m (Smith et al. 2013). The laboratory determined estimations for grout *K* ranged from 0.001 to 2.0 × 10^–12^ m/s, with the deeper samples being less permeable. Smith et al. (2013) indicate this could be due to some mixing inconsistencies but also note that the triaxial testing could not be loaded to in situ effective stresses and were instead conducted at a confining pressure of 20 kPa (corresponding to an in situ effective stress of approximately 20 m). For this reason, the determined *K* may be overestimated by up to an order of magnitude. It is also likely that the grout will continue to cure with time, resulting in a further decrease in *K* and increase in strength and stiffness (Devos and Raventos 2022). While this can be beneficial for very low *K* formations as seen in this study, it could also result in possible cracking and the creation of new hydraulic short circuits (Devos and Raventos 2022).Once in place, the transducers were connected to data loggers and initially programmed to measure pressure and temperature at 30 min intervals (Smith et al. 2013).

At site 2, a total of five boreholes were drilled using an Ingersoll Rand TH55 DH Top Drive hydraulic rotary drill rig between 2013 and 2014 within an 18 km^2^ area. Two boreholes were drilled 5 m apart to a total depth of 200 m each (site 2a). One of the boreholes was continuously cored (hereafter named corehole) and the other was drilled (hereafter named drillhole). Consistent with site 1, Geokon 4500S and 4500SH stainless steel VWPs were used at all five boreholes at site 2. Ten VWPs were installed at 20, 40, 60, 80, 100, 120, 140, 160, 180, and 200 m BG in the drillhole, and ten VWPs were installed at 10, 30, 50, 70, 90, 110, 130, 150, 170, and 190 m BG in the corehole, resulting in a VWP installation frequency interval of 10 m at this location. Three additional boreholes were continuously cored in the surrounding area, one to a total depth of 150 m BG with eight VWPs installed at 10, 30, 60, 80, 100, 120, 140, 150 (site 2b), and two to a total depth of 100 m with three VWPs installed at 30, 75, and 100 m BG (site 2c and 2 d). The only borehole that was not continuously cored was the drillhole at site 2a. All VWP installation procedures were identical to the procedures described at site 1. A barologger was installed at ground surface at each site to measure barometric changes at surface with the same time stamp as the VWPs. Long-term monitoring was completed by programming the dataloggers to record pore pressure, temperature, and barometric pressure at 30-min intervals between 2009 and 2025 at site 1 and 2013/14–2017 at site 2. A land ownership change at site 2 resulted in the decommissioning of all boreholes with installed VWPs; but at the time of disconnection, all instruments were working properly. In 2021, an attempt was made to splice the instrument cables and reconnect them to a datalogger, but it was unsuccessful as the data was sporadically erroneous.

### Analysis of pore pressure data

Identifying small changes in pore pressure due to barometric pressure is easiest if all other external events influencing pore pressure are minimal. For example, if the pore pressure is still recovering from the initial installation, the recovery will overshadow any small pore pressure changes (including those induced by barometric pressure). Similarly, if the transducers are located in a formation experiencing dynamic activity (e.g., dewatering or subsidence caused by mining or seismic activity), it can be difficult to sort through the data to find multiple periods of time where the pore pressure responses are primarily due to a known surface pressure change (i.e., barometric pressure). As such, installations in isolated areas with little surface activity provide a good baseline for natural pore pressure responses. In most cases, even at equilibrium, pore pressure will trend upwards or downwards to some degree. This slow trending (e.g. <  ~ 1 m H_2_O over 30 days) does not mask the pore pressure responses due to short-term barometric fluctuations and typically does not complicate the analysis.

#### Loading efficiency, compressibility, and specific storage

In a laterally homogeneous formation, stress changes caused by atmospheric pressure is aerially extensive and applies an almost entirely vertical load (or stress) to the ground surface. Horizontal displacement is considered negligible (Van der Kamp and Gale 1983). It is well established that pore pressure fluctuations as measured by grouted-in piezometers respond directly to barometric fluctuations. Traditionally, to convert the absolute pressure measured by VWPs to a gauge pressure, the barometric pressure at the time of installation is first subtracted from the pore pressure. In other words, the VWP is set to zero and works under the assumption that the barometric pressure at time 0 (*T* = 0) is representative of the average barometric pressure at the site. To remove this assumption, as was done in the current study at both sites and described by Smith et al. (2013), the average barometric pressure of all barometric measurements recorded was used in place of the barometric pressure at *T* = 0. The barometric fluctuations influencing the pore pressure record during monitoring is removed by subtracting the change in barometric pressure (*B* – *B*_ave_, where *B* is the barometric pressure at some time after *T* = 0 and *B*_ave_ is the average barometric pressure over the monitoring period). This standard correction inherently accepts that the entire change in vertical stress (or load) is borne by the pore water (i.e., loading efficiency, *γ* = 1). However, in overconsolidated argillaceous formations such as those examined in this study, the “stiffness” of the formation may allow a portion of the load to be supported by the solid matrix, thereby reducing *γ* (i.e., < 1). Therefore, the pore pressure response to known surface pressure changes, such as barometric pressure, is a result of the stiffness of the formation relative to the pore water as reflected by the formation’s *γ*. Thus, the one-dimensional (1-D) constrained compressibility (*m*_*v*_) of the formation can be determined by evaluating the proportion of the load borne by the pore water vs. the proportion of the load borne by the formation soil matrix.

The *γ* of the aquitard was estimated by analyzing pore pressure responses to known barometric fluctuations following the method by van der Kamp and Schmidt (1997) and described in detail by Smith et al. (2013). While transient effects immediately following installation can influence the magnitude of the barometric response, the *γ* analysis in this study was conducted only after pore pressures had stabilized, minimizing the influence of short-term installation-related transients. Because barometric loading applies an aerially extensive vertical stress change, all units in the profile experience essentially the same imposed load, resulting in minimal transient vertical gradients along the borehole and reducing the likelihood that the issues noted for transient FGM installations would affect the *γ* determinations.

Changes in barometric pressure (*B* – *B*_ave_) were systematically multiplied by values between 0 and 1.0 (representing *γ*) and subtracted from the raw pore-water pressure data. The resulting trends were superimposed to visually identify the value of *γ* that best minimized the fluctuations between barometric events. Once *γ* was established, values of *m*_v_ and specific storage (*S*_S_) were calculated using Eqs. 1 and 2, respectively:

1 $${m}_{\mathrm{v}}= \frac{\gamma {n}_{\mathrm{T}}\beta }{1-\gamma }$$

where β is the bulk compressibility of water (4.6 × 10^–7^ kPa^−1^), and *n*_T_ is total porosity.

*S*_S_ was then calculated using:

2 $${\mathrm{S}}_{\mathrm{S}}=\rho g\left({n}_{\mathrm{T}}\upbeta +{m}_{\mathrm{v}}\right)$$

where *ρg* is the unit weight of water (9.8 kN m^−3^).

Bulk compressibility (*α*) was calculated from *m*_*v*_ using the relationships outlined in (Duncan and Bursey 2013) using Poisson’s ratios based on reported values of claystones, cement, and glacial till (0.33, 0.2, and 0.3, respectively) (Gercek 2007; Klohn 1965).

#### Recovery modeling for K_h_

VWPs installed using the FGM typically produce a distinct pore pressure response following installation. Once the instrument is positioned in the borehole and grouting begins, pore pressure rises rapidly and peaks when grouting is complete. This peak corresponds to the pressure exerted by the grout column, which is a function of grout density and installation depth (Smith et al. 2016). As the grout cures, water is consumed through an exothermic reaction, resulting in a temperature increase and subsequent decline in pore pressure to near zero. The low water content in the grout creates a hydraulic gradient between the grout annulus and the adjacent formation allowing water to seep into the annular space and initiate a pore pressure recovery that gradually approaches the far-field pore pressure at that depth. The rate of pore pressure recovery is theoretically influenced by the *α* and *K* of both the formation and the grout. For approximately 10 days post-installation, the pressure signal may be influenced by thermal, chemical, and mechanical disturbances. Beyond this period, recovery is primarily driven by fluid flow from the formation and is therefore most sensitive to the formations *α* and *K*_h_ (Smith et al. 2016).

The in situ *K*_h_ at each VWP depth was estimated by simulating pore pressure recovery following VWP installation using a 2D axisymmetric, finite element numerical model SEEP/W, as described in Smerdon et al. (2014) and Smith et al. (2016). The best fit simulations were compared to observed recovery data using the root mean square error model verification as described in Smith et al. (2016). Grout properties were characterized using laboratory determined values for total porosity (*n*_T_) and *α* values for the grout at site 1 (Smith et al. 2013), and site 2a, while formation compressibility at each VWP location was determined using the loading efficiency method described in the “Loading efficiency, compressibility, and specific storage” section. Once pore pressure stabilized, the far-field hydraulic head was defined and used as a boundary condition in the numerical model. The long-term recovery trend was then simulated to identify the in situ* K*_h_ value that best matched the observed data. Further details on model calibration and sensitivity analysis are provided in Smith et al. (2016).

## Results and discussion

### Pore pressure analysis

Prior to VWP installation, the instruments were tested at the surface to confirm they were all operational following the procedure outlined in the instruction manual. Of the 34 VWPs installed at site 2, only one VWP was unresponsive post-installation (site 2c at 100 m BG). The most probable cause of failure is mechanical damage, either from impact against the borehole wall or pinching of the cable during installation. All ten VWPs installed at site 1 were functioning properly post-installation. The successfully installed VWPs remain operational after 17 years at site 1, but the site 2 boreholes were decommissioned after 3–4 years of monitoring.

**Fig. 2:**
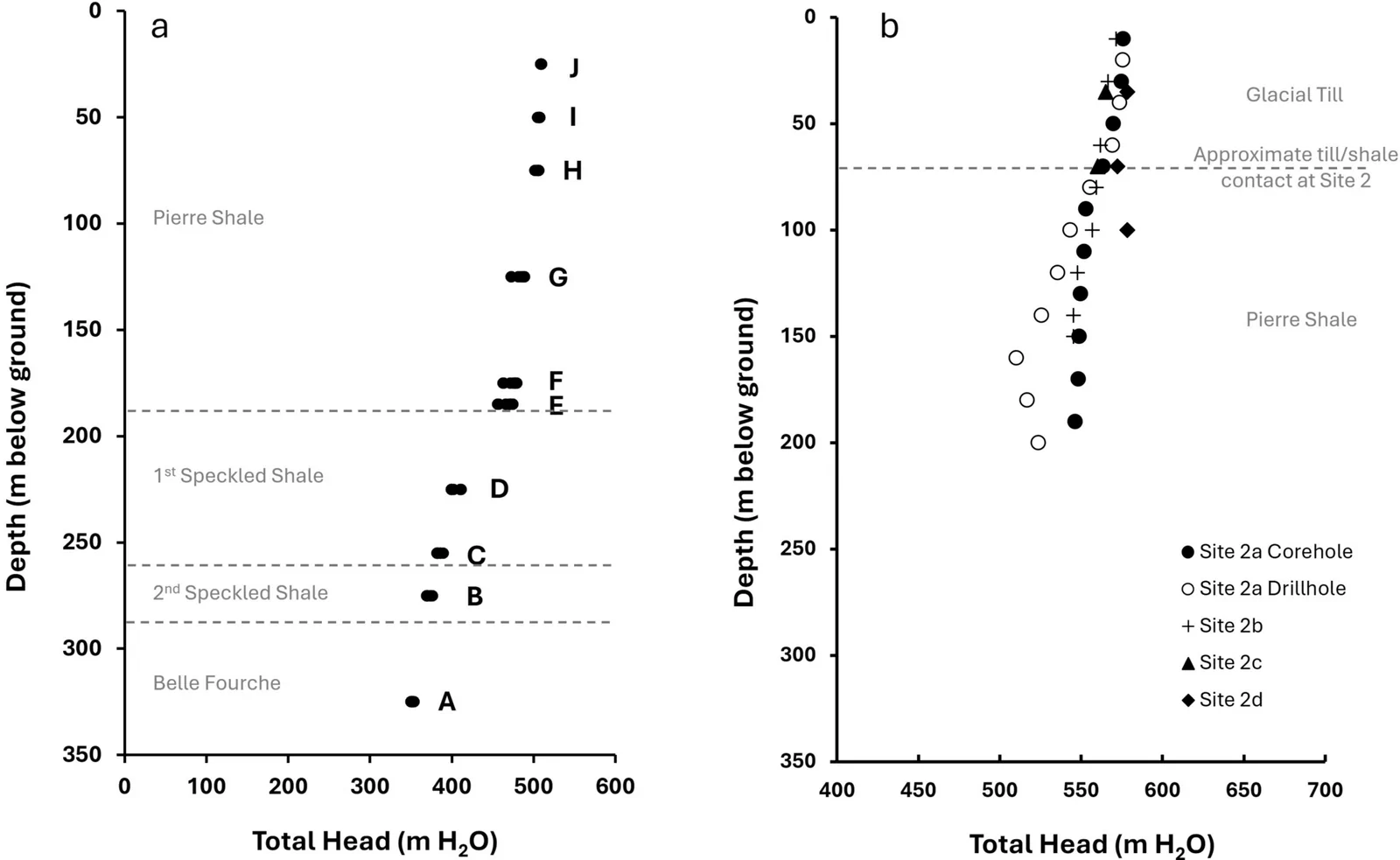
Total hydraulic head profiles at (**a**) Site 1 and (**b**) Site 2. Site 1 profiles show annual head measurements over 16 years, with notable variability in transducers D-G. Site 2 profiles represent conditions at the end of monitoring in 2017 (after 3–4 years of monitoring). The VWPs installed below 100 m depth in the drillhole had still not fully recovered at the end of the monitoring period

However, the VWPs installed below 80 m depth in the drillhole at site 2a did not fully recover over the 3-year monitoring period. Typically, pore pressure stabilizes within about 6 months depending on the *K*_h_ of the adjacent formation, so this observation was unexpected. The incomplete recovery is attributed to differences in drilling techniques (i.e., rotary drilling vs continuously coring) which may have resulted in varying degrees of borehole wall disturbance. The rotary-drilled borehole may have developed a more pronounced skin effect, potentially due to the accumulation of drilling fluid with high bentonite content and drill cuttings along the borehole annulus, thereby impeding connectivity with the surrounding formation. The mechanics responsible for this behavior are currently being investigated in a separate manuscript given the importance of VWP installation techniques for reliable in situ pore pressure measurements. For the purposes of this study, all VWPs installed below 80 m depth in the drillhole at site 2a have been excluded from the results (apart from the total head profile in Fig. 2) as the *γ* method cannot be used to reliably determine *γ* from pore pressure data showing continued transient fluctuations. However, the VWPs installed at 20, 40, 60, and 80 m BG in the drillhole did stabilize in the expected timeframe and are included in the results.

The water table at both sites is near the surface and fluctuates between 1 and 3 m seasonally at site 1 (Hendry et al. 2013). Figure 2 illustrates the yearly total head profiles with depth at site 1 (Fig. 2a) and site 2a, b, c, and d (Fig. 2b). The shallowest VWPs at both sites (J, I, and H at site 1, and all transducers installed above 60 m at site 2) are consistent with hydrostatic pressure. There is a downward hydraulic gradient of 0.2 and 0.11 through the Pierre Shale at site 1 and site 2, respectively. At site 1, the VWPs installed in the lower portion of the Pierre Shale (transducers G, F, and E), show the largest changes in pore pressure over the 16-year monitoring period (as shown by the range of values in Fig. 2a at these depths). Transducer D also shows a comparatively large range, though this behavior is likely not related to the behavior causing the larger range in the Pierre Shale (discussed in the “Long-term performance and data continuity of VWPs” section). It has been established that the VWPs installed below a 5 m thick bentonite layer at the top of the 1 st Speckled Shale is likely hydraulically isolating the VWPs above the Pierre Shale/1st Speckled Shale contact (Smith et al. 2016).

#### Loading efficiency

**Fig. 3:**
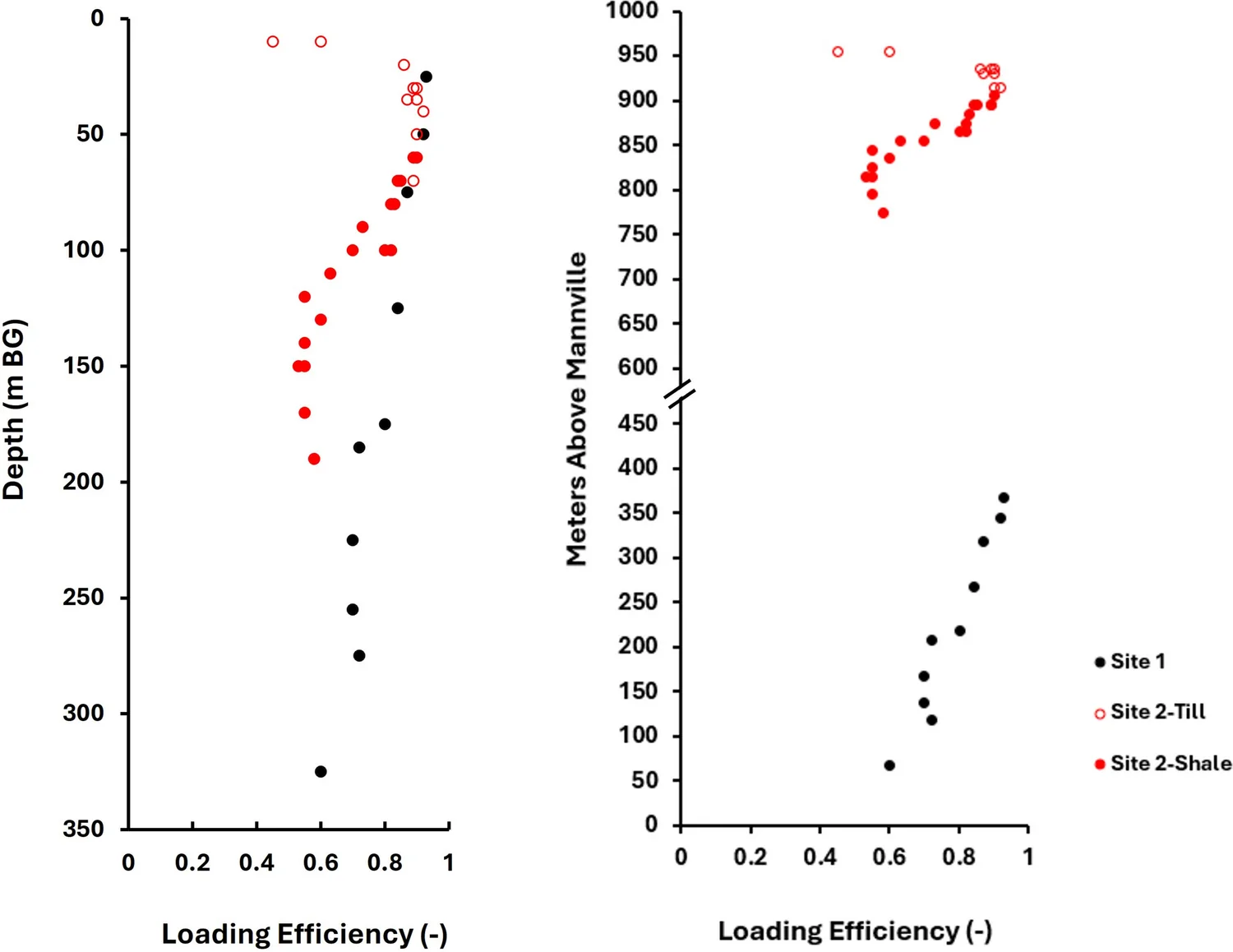
Loading efficiency (*γ*) values determined from VWPs installed in shale at site 1 (black circles), and in glacial till (open red circles) and shale (red circles) at site 2. Results are plotted by (**a**) depth below ground surface and (**b**) elevation above the Mannville Formation, a regionally extensive aquifer

The *γ* at the location of each VWP was determined following pore pressure stabilization (i.e., 3–6 months after installation) at all sites and once again near the end of the monitoring periods following the method described in the “Loading efficiency, compressibility, and specific storage” section (Fig. 3). The results are presented both with depth (a) and as m above the Mannville formation (b) as it is a regionally extensive formation present at both sites. The initial *γ* determined for each location was not referenced prior to redetermining the *γ* at the end of the monitoring period at each site to reduce possible bias in the interpretation. The difference between the *γ* determined at the beginning of the monitoring period and at the end of the monitoring period at both locations was negligible (< 0.02) and indicates the *γ* was stable throughout the monitoring period at both sites. The similar *γ*, and thus formation *α*, is consistent with the expected stable geologic and groundwater conditions at both sites.

##### Shale

At site 1, *γ* decreased with depth, ranging from approximately 0.6 near the bottom (325 m BG) of the borehole to 0.93 near the top of the borehole (25 m BG). The reduction in *γ* with depth is attributed to increasing consolidation of the deeper geologic material. As a result, a greater proportion of the applied vertical stress can be borne by the soil skeleton and transferred from the pore water, thereby reducing the *γ* with depth (Smith et al. 2013). The VWPs at this location are installed throughout three formations, the Pierre Shale (11–184 m BG), the First and Second Speckled Shale (184–281 m BG), and the Belle Fourche (281–336 m BG) formations. Despite some localized differences in the geotechnical and hydrogeological parameters between these formations (Smith et al. 2013, 2016; 2018), *γ* decreases roughly linear with depth (R^2^ = 0.85). This would suggest that the “stiffness” of the formation is the dominant control on the resulting *γ* and is further supported by the decreasing trend observed from 1D consolidation tests completed on core samples from this location (Smith et al. 2018).

The VWPs installed in the Pierre shale at site 2 show a similarly decreasing trend with depth, consistent with observations at site 1. This trend is also likely due to increasing consolidation with depth. However, notable differences in *γ* vs depth are observed between the two sites, particularly between 125 and 185 m BG (Fig. 3a). The two sites are separated by up to 350 km, and differences in the evolution of the WB at each location (i.e., thicknesses of overburden, glacial loading/unloading, erosion) may influence the formational stress history, and thus the determined *γ* with depth. However, the overall decreasing trend is attributed to increasing effective stress (*σ*′) with depth. These factors could explain why the shale at site 2 appears more consolidated at shallower depths compared to site 1. Further, unlike at site 1, the *γ* does not appear to decrease linearly through the entire profile (Fig. 3). Near the top of the shale at site 2, the *γ* is approximately 0.9 then decreases roughly linearly until around 140 m BG where the *γ* stabilizes between 0.55 and 0.58 until 200 m BG. A *γ* of 0.55 corresponds to a bulk compressibility of 3 × 10^–7^ kPa^−1^, which is slightly lower than the compressibility of water (*β* = 4.7 × 10^–7^ kPa^−1^). The lowest *γ* values observed in argillaceous formations using the *γ* method is around 0.5, suggesting there may be a practical lower limit for *γ* estimation. This does not imply that the *γ* method is unsuitable for stiffer materials that may yield lower *γ* values but rather indicates that there may be a natural lower bound of *α* in argillaceous formations that approaches the compressibility of water.

##### Glacial till

At site 2, the *γ* determined from the shallowest VWPs located in till formations were uncharacteristically low for glacial till. Typically, till is more compressible than shale due to its lower degree of consolidation and greater heterogeneity. However, the two VWPs located at 10 m BG at site 2a and 2b resulted in *γ* estimates of 0.6 and 0.45, respectively, and suggest that the upper 10 m of the till is unusually stiff. This stiffness could be due to the heterogeneous nature of glacial tills and exacerbated by near surface weathering and repeated freeze–thaw cycles, which can lead to the formation of a dense, hard layer resembling a hardpan. In contrast, the VWPs installed at greater depths in the till (20–50 m BG) yielded more typical *γ* values for till formations, ranging from 0.85 to 0.92. At these shallow depths, the unusually low *γ* values may also reflect vertical flow within the borehole if a permeability contrast existed between the grout and the surrounding till, or differential stiffness between the grout column and the formation that could alter the barometric response. While the depth-dependent variability in *γ* suggests that complete vertical short-circuiting is unlikely and the grout to formation permeability is expected to be within the acceptable range, these mechanisms represent plausible contributing factors and are acknowledged as alternative explanations for the behavior observed at 10 m. These findings highlight the importance of delineating in situ compressibility in glacial tills, particularly in heavily glaciated regions where infrastructure and mining are frequent and subsurface variability can influence design and performance.

##### Fractured shale

Three VWPs were installed in the First and Second Speckled Shale at site 1, which is known to have polygonal fracturing throughout (Smith et al. 2016). Despite the fracturing, the estimated *K*_*h*_ remains low, on the order of approximately 10^–11^ m/s (Smith et al. 2016), which suggests that many of the fractures may be partially or fully infilled with secondary minerals or fine-grained material, or that self-healing processes, such as swelling of clay minerals, have reduced their permeability over time. As a result, the overall contribution of these fractures to groundwater flow may be somewhat limited. The pore pressure responses recorded by the VWPs installed in the fractured shale formations yielded *γ* values from 0.6 to 0.7 and indicate that the stiffness of the formation remains the dominant factor in resulting *γ* determinations. However, since only three VWPs were installed in fractured shale at a single location, additional measurements from other fractured shale formations are necessary to strengthen confidence in the observed values and ensure broader applicability. Notably, the application of VWPs in fractured materials may represent one of the most valuable applications of these in situ methods, since fractured core samples are not generally competent enough for reliable laboratory testing. As a result, these formations are often excluded from testing altogether and hydrogeologic parameters are assigned on the basis of a broad generalized range. In some cases, the presence of natural fractures would be overlooked because observed fractures in the core are mistakenly attributed to drilling-induced damage and more competent core samples from above or below the fractured zone are used as a proxy for the damaged sample zones.

#### Compressibility and specific storage

**Fig. 4:**
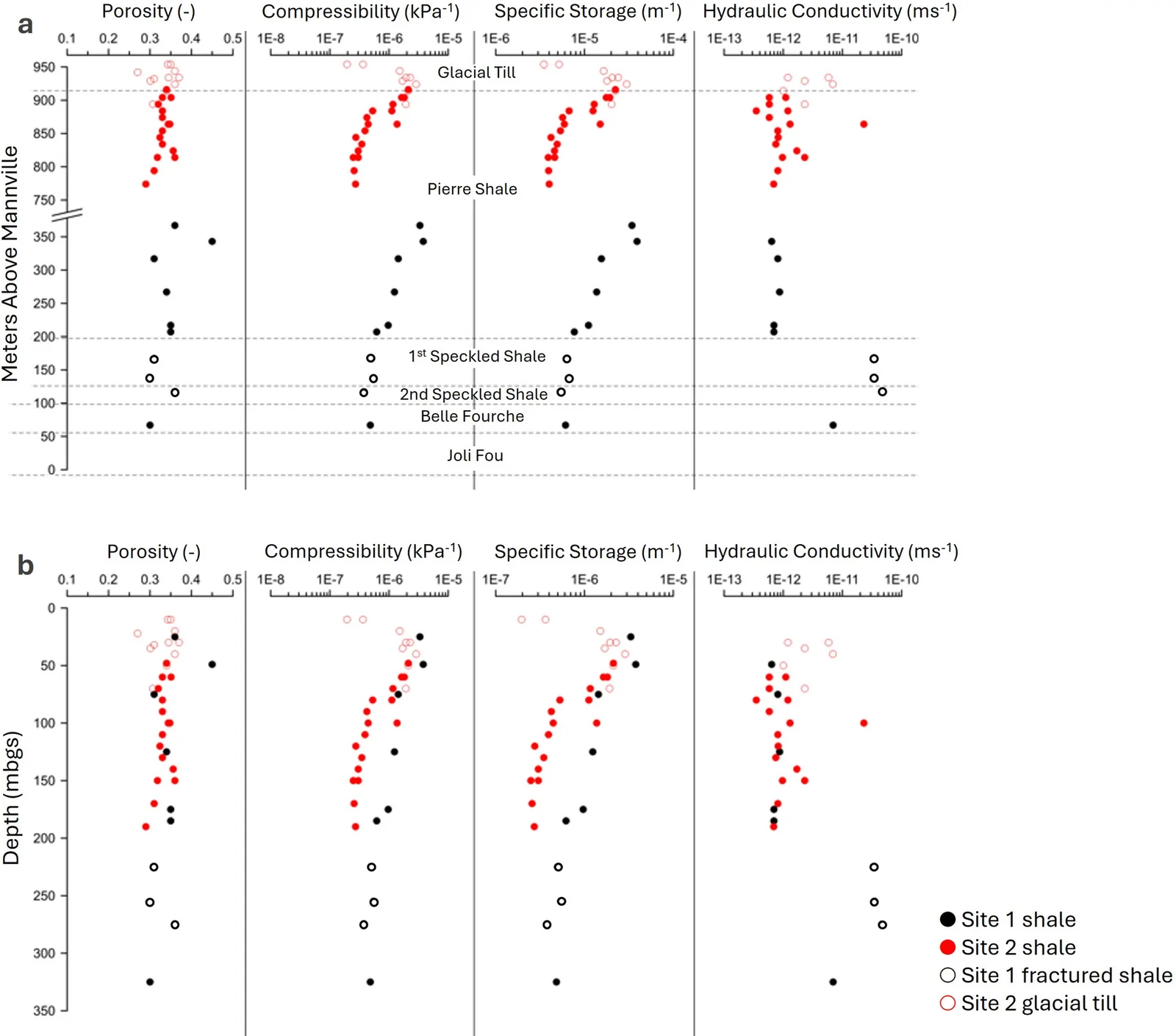
Comparison of laboratory determined porosity and VWP-derived in situ estimates of *α*, *S*_S_, and *K*_h_*.* Results are plotted by (**a**) elevation above the Mannville Formation and (**b**) depth below ground surface. A break in the vertical axis in (**a**) removes 400 m of Pierre Shale where no transducers were installed. Closed circles represent the VWPs installed in the shale formations at site 1 (black) and site 2 (red). Open circles represent the VWPs installed in the fractured shale at site 1 (black, open), and glacial till at site 2 (red, open)

The *m*_v_ and *S*_S_ at the location of each VWP installed at site 1 and site 2 were calculated using Eqs. 1 and 2. Bulk compressibility (*α*) was calculated on the basis of the bulk modulus (1/B) following Duncan and Bursey (2013). With the exception of the two VWPs installed 10 m at site 2, *α* and *S*_S_ generally decrease with increasing depth similar to the *γ* trend which is expected given the relationships described in Eqs. 1 and 2 (Fig. 4, Table 1). A full examination of laboratory determined *α* vs VWP determined *α* in the context of stress behavior observed in laboratory tests and its implications for in situ conditions is provided in Smith et al. (2013, 2018).

**Table 1:** Summary of *S*_S_ and *K*_h_ values determined at the location of each transducer using the loading efficiency method at site 2. Ranges from site 1 are included for comparison, based on previously published data (Smith et al. 2013, 2016)

	Lithology	Depth (m)	Porosity (–)	Specific storage (m^−1^)	Horizontal hydraulic conductivity (ms^−1^)
Site 2 corehole	Glacial till	10	0.35	3.94 × 10^–6^	-
	Glacial till	30	0.37	1.67 × 10^–5^	1.2 × 10^–12^
	Glacial till	50	0.34	1.53 × 10^–5^	1.0 × 10^–12^
	Pierre shale	70	0.32	9.02 × 10^–6^	5.8 × 10^–13^
	Pierre shale	90	0.33	5.51 × 10^–6^	5.8 × 10^–13^
	Pierre shale	110	0.33	4.02 × 10^–6^	8.1 × 10^–13^
	Pierre shale	130	0.33	3.72 × 10^–6^	7.5 × 10^–13^
	Pierre shale	150	0.36	3.61 × 10^–6^	9.7 × 10^–13^
	Pierre shale	170	0.31	3.11 × 10^–6^	8.1 × 10^–13^
	Pierre shale	190	0.29	3.11 × 10^–6^	6.9 × 10^–13^
Site 2 drillhole	Glacial till	20	0.36	1.16 × 10^–5^	-
	Glacial till	40	0.36	2.03 × 10^–5^	6.9 × 10^–12^
	Pierre shale	60	0.33	1.35 × 10^–5^	5.8 × 10^–13^
	Pierre shale	80	0.33	8.26 × 10^–6^	3.5 × 10^–13^
	Pierre shale	100	0.33	-	-
	Pierre shale	120	0.33	-	-
	Pierre shale	140	0.36	-	-
	Pierre shale	160	0.31	-	-
	Pierre shale	180	0.29	-	-
	Pierre shale	200	0.30	-	-
Site 2a	Glacial till	10	0.34	2.81 × 10^–6^	-
	Glacial till	30	0.35	1.41 × 10^–5^	5.8 × 10^–12^
	Pierre shale	60	0.35	1.58 × 10^–5^	1.1 × 10^–12^
	Pierre shale	80	0.33	8.75 × 10^–6^	1.2 × 10^–12^
	Pierre shale	100	0.34	8.62 × 10^–6^	1.3 × 10^–12^
	Pierre shale	120	0.32	3.25 × 10^–6^	8.2 × 10^–13^
	Pierre shale	140	0.36	3.57 × 10^–6^	1.7 × 10^–12^
	Pierre shale	150	0.32	3.05 × 10^–6^	2.3 × 10^–12^
Site 2b	Glacial till	35	0.34	1.50 × 10^–5^	-
	Pierre shale	70	0.37	1.10 × 10^–5^	-
	Pierre shale	100	0.33	-	-
Site 2c	Glacial till	35	0.30	1.04 × 10^–5^	2.3 × 10^–12^
	Glacial till	70	0.31	1.26 × 10^–5^	2.3 × 10^–12^
	Pierre shale	100	0.35	7.84 × 10^–6^	2.3 × 10^–11^
Site 2	Glacial till			0.281–2.03 × 10^–5^	1.2–6.9 × 10^–12^
	Shale			0.305–1.58 × 10^–5^	0.35–2.3 × 10^–12^
Site 1 ^a^	Shale			0.451–2.56 × 10^–5^	0.63–6.9 × 10^–12^
	Fractured shale			4.06–4.83 × 10^–6^	0.35–4.6 × 10^–11^

^a^ Smith et al. (2013, 2016)

A general relationship between *n*_T_ and *S*_S_ for normally to overconsolidated argillaceous media was defined by Konikow and Neuzil (2007) using results from laboratory tests of various formations reported in the literature and identified an expected range of *S*_S_ for different porosities (Domenico and Mifflin 1965; Skempton 1969; Cripps and Taylor 1981; Tellam and Lloyd 1981; and Neuzil 1993). The majority of the *S*_S_ data used to define this relationship was based on laboratory determined *α* values; therefore, it is reasonable to conclude that *S*_S_ would decrease with decreasing *n*_T_ because it is related to a decrease in porous matrix *α* with compaction (Konikow and Neuzil 2007). Smith et al. (2013) revisited this relationship and added the results of the transducer determined *S*_*S*_ data from site 1. A number of the in situ *S*_S_ results plotted above the expected range for overconsolidated material, suggesting the upper boundary should be extended upward to include in situ estimates of *S*_S_. Consistent with the site 1 data, the in situ* S*_S_ estimates from site 2 predominantly plotted above the expected range indicated by (Konikow and Neuzil 2007).

**Fig. 5:**
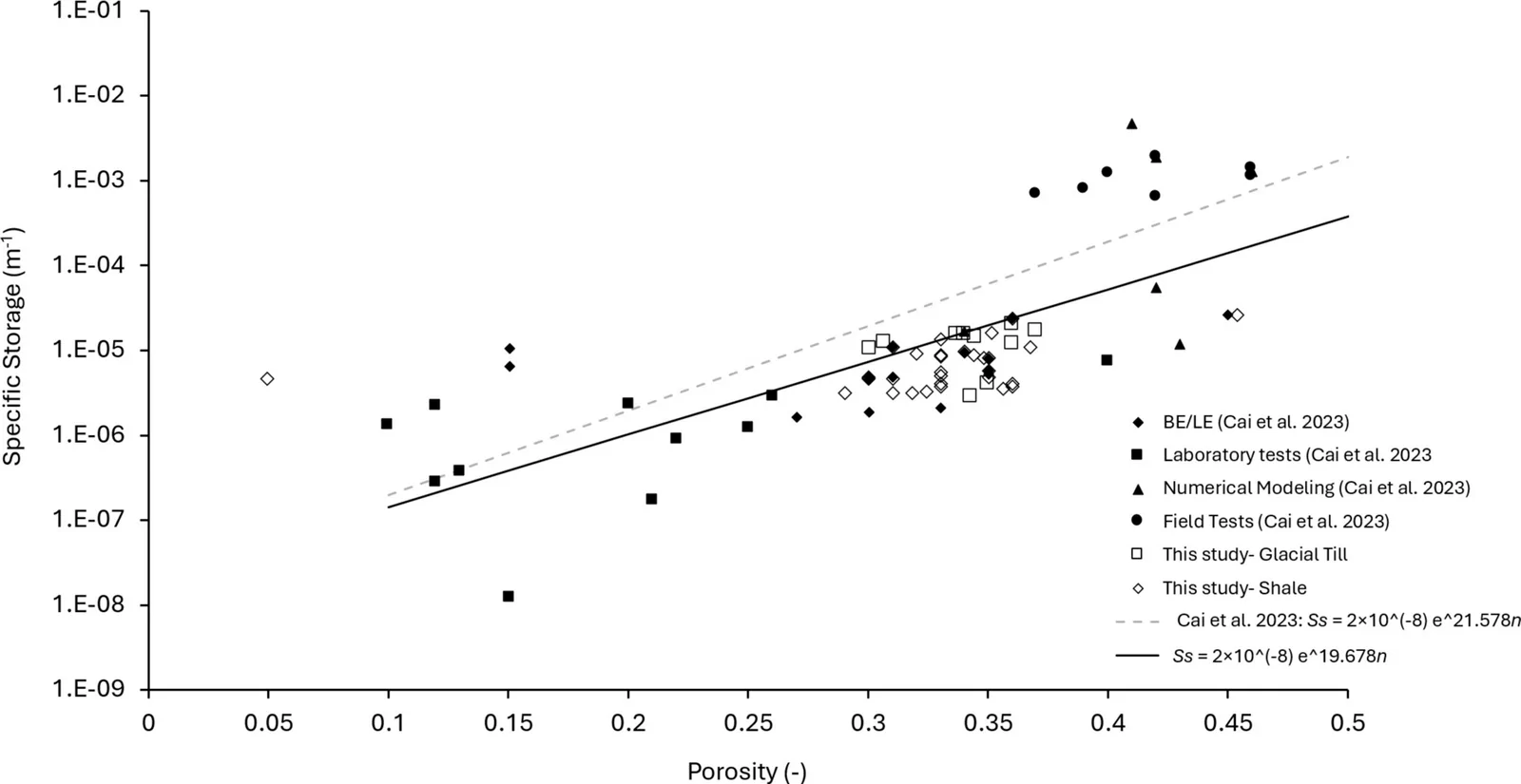
Relationship between *S*_S_ and porosity for argillaceous formations. Literature values (solid symbols) derived from laboratory, numerical modeling, and field methods include the exponential trend reported by Cai et al. (2023) (dashed line). In situ VWP-derived estimates from this study and Smith et al. (2013, 2016; open symbols) were added to the dataset, resulting in a revised trend line (solid line)

More recently, Cai et al. (2023) compiled specific storage data from the literature for a range of aquitard types worldwide using various methods, including laboratory testing, field investigations (e.g., slug tests, pumping tests, barometric/loading efficiency methods), and numerical modeling (Fig. 5). When plotted against porosity, the data showed a positive exponential relationship, indicating the *S*_S_ increases with porosity (*n* = 43; *S*_S_ = 2 × 10^−8^e^22.29*n*^) (Cai et al. 2023). However, the original data set included non-argillaceous materials such as dolomite, granite, and silty sand. When these types of material were excluded and only data from shale, mudstone, glacial till, clay, claystone, and silty clay were considered, the relationship changed slightly to (*n* = 39; to *S*_S_ = 2 × 10^−8^e^21.578*n*^) as shown by the dashed line in Fig. 5 (Cai et al. 2023). Incorporating all the VWP derived *SS* values at site 1 and site 2 in this study further shifted the trend to *S*_S_ = 2 × 10^−8^e^19.678*n*^ as shown by the solid line in Fig. 5. Despite these changes, the overall exponential pattern remained consistent.

#### Hydraulic conductivity

**Fig. 6:**
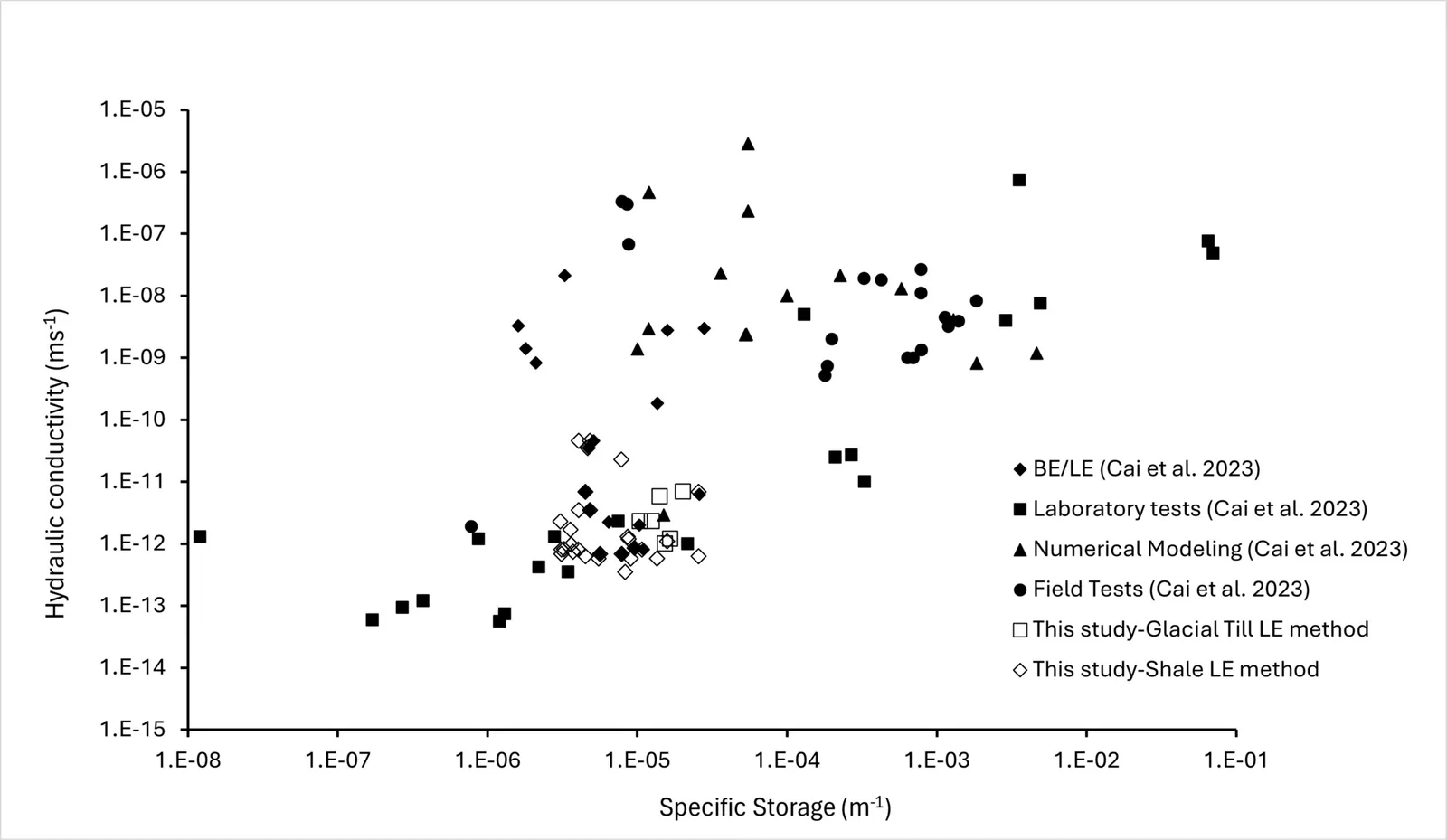
Relationship between *S*_S_ and *K* for argillaceous formations. Literature values derived from laboratory testing, numerical modeling, and field tests (closed symbols; Cai et al. 2023) are shown alongside in situ VWP-derived estimates from glacial till and shale at site 1 (Smith et al. 2016) and site 2 (this study) (open symbols)

The recovery method described in the “Recovery modeling for Kh” section was applied to the VWPs installed at site 2 using the same approach previously implemented at site 1 (Smith et al. 2016). The transducer determined results from the VWPs installed in the till formations at site 2 indicated *K*_h_ ranged from 1.2–6.9 × 10^–12^ ms^−1^ with the lower values observed near the top of the profile. This variation is consistent with the overconsolidated nature of the till as evidenced by the *γ* and *α* analysis discussed above. The transducer determined results from the VWPs installed in shale formations at site 2 ranged from 0.35 to 2.3 × 10^–12^ ms^−1^ and are consistent with estimates from the shallower VWPs (25–185 m BG) at site 1, which range from 0.63 to 6.9 × 10^–12^ ms^−1^ (Fig. 6). The deeper VWPs at site 1 are installed in shale formations with polygonal fracturing, resulting in a *K*_h_ approximately one order of magnitude greater than those in non-fractured shale (0.35–4.6 × 10^–11^ ms^−1^) (Smith et al. 2016). The results from this study are plotted along with values reported in the literature in a *K* vs *S*_S_ plot to identify general trends and assess if particular methods result in specific ranges (Fig. 6). While there appears to be a broad positive correlation between *K* and *S*_S_ in argillaceous formations, the scatter and variability across methods prevent the definition of an empirical relationship. Nonetheless, the plot highlights where the in situ estimates tend to plot relative to laboratory or modeling-based results.

Most reported *K* values for aquitards in the literature are derived from triaxial or odometer laboratory testing which typically yield estimates of vertical *K* (*K*_v_). While sample disturbance can introduce some degree of error, the primary concern with laboratory-determined *K* values are scale effects (van der Kamp 2001). If the core samples selected for testing are not representative of the formation (e.g., including heterogeneities or fractures), the resulting measurements reflect the matrix *K*, rather than the bulk *K* (van der Kamp 2001). This reasoning may explain the often-large discrepancies observed between field and laboratory-determined *K* values for the same materials (Bredehoeft et al. 1983). In situ estimates of *K*_h_ of aquitard formations are scarce in the literature, largely because conventional field methods can take months, if not years, to yield results due to slow response times of low-*K* materials. Those that do exist, often report much higher *K* values than other methods, likely because the tests were only feasible when macrofeatures (i.e., fractures) or heterogeneities (i.e., sand layers or lenses) permitted the testing to be successful in a reasonable amount of time.

On the basis of the current study and values reported in the literature, the horizontal *K*_h_ of argillaceous materials using the barometic/loading efficiency method spans a wide range, from approximately 1 × 10^–13^ to 1 × 10^–8^ ms^−1^. These values are generally lower than results obtained from numerical modeling and field tests. The laboratory results consistently yield the lowest *K* values, though they may not accurately reflect the in situ conditions if more competent core samples are preferentially selected for testing over fractured or heterogeneous samples. The wide range of *K* values obtained from the various methods highlights the heterogeneous nature of argillaceous materials and the challenges in characterizing the hydrogeological parameters of aquitard formations. This study reinforces that no single method can fully capture the hydraulic behavior of low-*K* permeability formations, and a multi-method approach is needed to accurately characterize these materials.

### Long-term performance and data continuity of VWPs

**Fig. 7:**
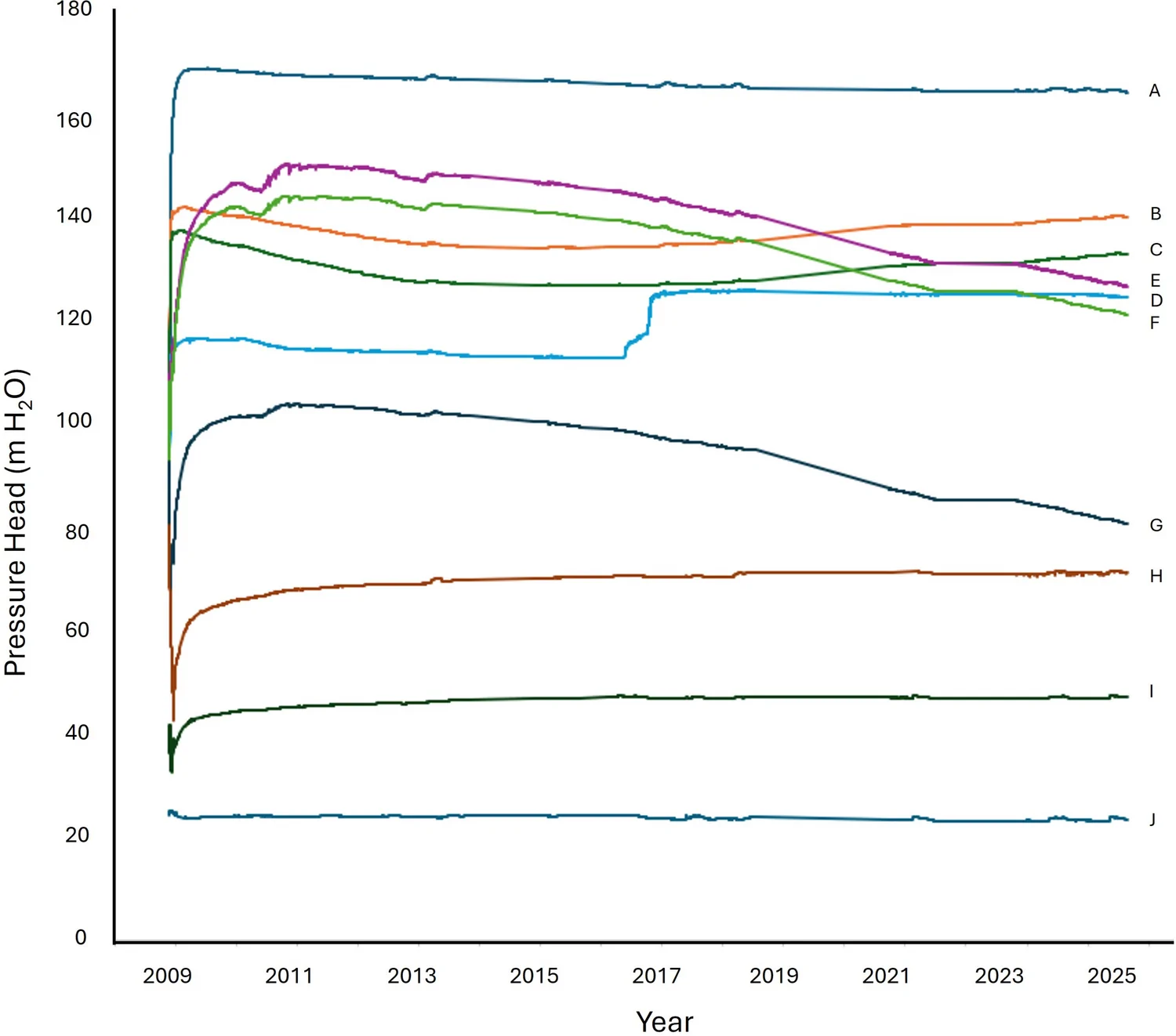
Long-term pressure head (m H_2_O) recorded by ten VWPs installed at site 1, spanning depths from 25 m (transducer J) to 325 m (transducer A). Data illustrate stable, declining, and anomalous trends over a 16-year monitoring period

The VWPs installed at site 1 have been recording since installation in 2009. Over the past two decades, various circumstances have interrupted recording of pore pressure measurements, including theft of solar panels on multiple occasions, modem and telemetry updates, and restricted access due to weather or surface work. However, no other study appears to have reported near-continuous long-term pore pressure of a thick aquitard sequence such as the one presented here. VWPs are expected to function for 10–20+ years when installed properly, and to date, all VWPs installed at site 1 are functioning after 16 years (Fig. 7). However, it does appear that transducer H (75 m BG) is beginning to show some anomalous results starting in 2023 as illustrated by small periodic jumps in pore pressure and may indicate the instrument is starting to fail. Gaps in the data, most notably between mid-2019 and early 2021, were filled by connecting the trendline, though no data was added for these time periods.

The shallowest transducer J (25 m BG) has remained stable since the initial recovery after installation (< 1 m change) and is consistent with theoretical hydrostatic pressure at this location given the water table is 2–3 m below ground (Hendry et al. 2013) with a ground elevation of 510.3 m (508.6 ± 1 m). Transducers I (50 m BG), and H (75 m BG) have exhibited a gradual increase in pore pressure over the past 15 years by up to 4 m but are generally stable. Transducers E (185 m BG), F (175 m BG), and G (125 m BG) all seem to follow the same general trend. After an expected recovery period post-installation in 2009, all three experienced short-term anomalies of abrupt increases and decreases in pressure in 2010–2011. Following a brief stabilization phase, pore pressures at these depths began a consistent, gradual decline. Over the monitoring period, transducers E and F recorded a 15 m drop in pressure head, while G showed an 18 m decline which is a considerable change for a formation that is generally considered to be near hydraulic equilibrium. Notably, these three transducers are located closest to an approximately 5 m thick bentonite layer at the top of the 1 st Speckled Shale, which hydraulically isolates the Pierre Shale from deeper formations (Smith et al. 2016). This isolation may explain why the VWPs below the bentonite did not exhibit similar pressure fluctuations in 2010 or show a large decline over the past 15 years. Interestingly, while E and F both exhibited the largest abrupt pore pressure changes in 2010, transducer G, located furthest from the bentonite layer, appears to exhibit a dampened response to these effects, which suggests that the source of this behavior is occurring near the bentonite layer. The cause of these anomalies remains unclear, but the processes causing the behavior seem to disproportionately affect the zone above the bentonite, which may explain why only the VWPs installed at these depths are impacted.

Transducer D (225 m BG) appeared to stabilize following the initial recovery period, maintaining consistent readings of 113 m of pressure head until April 2017. Over the next 6 months, the pressure head increased by 12 m at this location. No other VWP at site 1 exhibited similar behavior during this time. Despite the anomaly, the transducer continues to respond to barometric fluctuations, suggesting it remains operational. Since the end of 2017, pore pressures have restabilized at approximately 125 m H_2_O, 12 m higher than those recorded between 2010 and 2016.

Transducers B (275 m BG) and C (255 m BG), positioned on either side of the 1 st and 2nd Speckled Shale contact, exhibit similar behavior to one another. Following initial stabilization, both recorded a decline in pore pressure of 4–5 m over a 5-year period. This was followed by a gradual increase over the subsequent 5 years, ultimately returning to their original stabilized pressure levels. Transducer A (325 m BG), the only VWP installed in the Belle Fourche Formation, has recorded slowly declining pore pressure over the monitoring period of about 3 m H_2_O.

The long-term performance of the VWPs installed at site 1 demonstrate the durability and reliability of VWPs in deep, low-*K* formations. Despite intermittent data gaps, the dataset represents one of the most comprehensive near-continuous pore pressure records for a thick aquitard sequence reported to date. The pore pressure responses observed across the profile reveal a range of behaviors, from stable hydrostatic conditions in the shallowest transducer (J) to more complex trends at intermediate and greater depths. Gradual increases in pore pressure at 50–75 m (transducers I and H) may indicate slow recharge is occurring at this depth. In contrast, the considerable declines observed between 125 and 185 m BG (transducers G, F, E) may indicate that localized hydraulic or mechanical influences are affecting this interval. The isolated pressure rise at 225 m BG (transducer D) in 2017 remains unexplained but does not appear to reflect instrument failure. The behavior between 255 and 325 m BG (transducers C, B, and A) may suggest that formation scale processes may be influencing pressure dynamics at depth.

The variation in pressure behavior with depth provides confidence that vertical short-circuiting through the grout annulus is not occurring. The VWPs appear to be independently responding to transient groundwater conditions at their respective depths. Overall, the data from site 1 provide valuable insight into the long-term hydraulic behavior of a thick aquitard system and highlight the importance of sustained monitoring to capture both gradual trends and short-term anomalies that may otherwise go undetected.

### Application to different lithologies

**Fig. 8:**
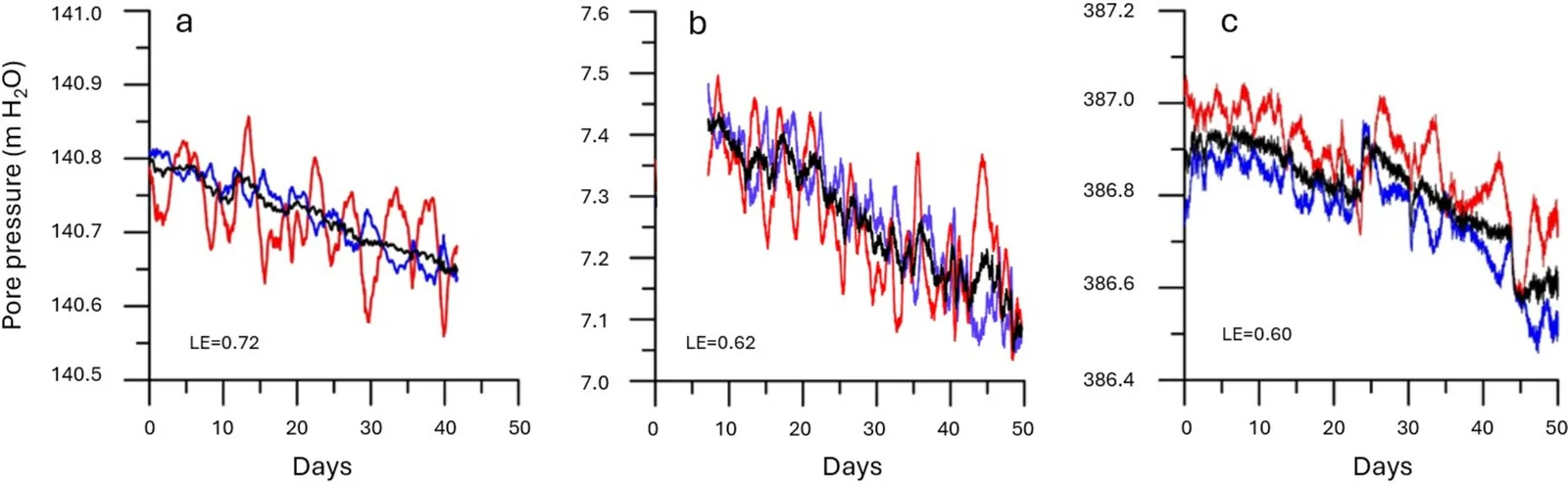
Example of pore pressure corrections using the *γ* method for (a) shale, (b) glacial till, and (c) fractured shale. The red trend is the pore pressure prior to application of the *γ* method, the blue trend is the “standard” barometric correction assuming *γ* = 1, and the black trend is the corrected pore pressure after the application of the *γ* method

VWPs installed at site 1 and site 2 were categorized into three lithological groups based on their installation depth and surrounding material: (a) shale (*n* = 31), (b) glacial till (*n* = 10), and (c) fractured shale (*n* = 3). The pore pressure responses for each material type are presented in Fig. 8. VWPs installed in overconsolidated shale formations typically exhibit the most stable and interpretable trends, with corrected pore pressure data showing smooth transitions in response to barometric pressure fluctuations and other external forcings (i.e., natural changes in pore pressure, or variations due to mining or seismic activity) (Fig. 8a). The small perturbations still present in the pore pressure record are likely due to other minor influences on the formation pore pressure (e.g., earth tides).

The 10 VWPs installed in glacial till at site 2 generally responded well to barometric pressure fluctuations (Fig. 8b); however, the corrected pore pressure trends showed more variability compared to those observed in shale. This difference could be attributed to differences in the geotechnical properties of the two materials and the heterogeneous nature of glacial tills. Glacial tills are also typically more compressible and heterogeneous than overconsolidated shale, which tends to be stiffer and more uniform. As a result, the correction of pore pressure in till formations may be influenced by spatial variability in many parameters, including *K*, *α*, *n*_T_, or drainage conditions. Nevertheless, the clear response in pore pressure to barometric fluctuations in till, along with the reduction of the highs and lows of the barometric effects indicate that the *γ* can be characterized by the application of the *γ* method. The pore pressure recovery analysis of the VWPs installed in the till formations at site 2 was successful, including the VWPs installed in the till at site 2a (drillhole), where the shale formations did not exhibit the expected recovery behavior. This observation not only reinforces the reliability of the recovery method for VWPs installed in glacial till but also offers insight into why the drilling method did not appear to affect the pore pressure recovery of the tills, but did influence the pore pressure recovery behavior in the shale.

At site 1, only three VWPs were installed in the fractured shale, so it is not possible to define a representative pore-pressure trend. The trends presented simply illustrate the types of pore pressure responses that may occur in this type of formation. The presence of fractures did not appear to influence the *γ* method as the pore pressures at both sites responded well to barometric pressure fluctuations (Fig. 8c). The recovery analysis from the VWPs in the fractured shale at site 1 was successful and similar to non-fractured shale or till formations, suggesting that both the *γ* and the recovery method are successful in fractured shale.

The repeatability of the VWP methods in various geologic media provides some confidence that the transducer-based method is a viable option for determining *K*_h_ and *α* in shale and till formations. However, using VWPs to determine in situ parameters should not eliminate the use of laboratory testing. A comparison of laboratory and in situ results provides information on the heterogeneity of the formation. If the laboratory results and the transducer results are different (e.g., the transducer results are 1–2 orders of magnitude greater than the laboratory results), these data could suggest that the laboratory scale tests are measuring the matrix *K* and not including the effects of secondary features (e.g., fractures). As such, a comparison of both methods could help determine if hydrogeologic heterogeneities exist in the formation (Smith et al. 2016) and better characterize local and regional groundwater flow.

Although consistency does not inherently imply correctness, it appears that both the *γ* method to determine *m*_*v*_ (and subsequently *α* and *S*_S_), and the recovery method to determine *K*_h_ are most successful in argillaceous shale formations. The application of the *γ* method in shale formations produces the “smoothest” corrected trend of any geology studied thus far and the pore pressure recovery following installation in shale formations consistently follows the characteristic recovery trend required to apply the recovery method and determine *K*_h_. A total of 44 VWPs were installed in Cretaceous shale and glacial till formations at sites 1 and 2, and only one VWP did not record pore pressure immediately following installation. This apparently faulty VWP measured correct pore pressure at surface but repeated –99999.99 (error) when connected to the datalogger after installation, thus suggesting the VWP was damaged during installation, and not due to the geology of the formation.

## Conclusions

This study demonstrates the long-term performance and application of fully grouted VWPs for characterizing the in situ hydrogeologic properties of argillaceous aquitards. Ten VWPs were installed in a 325 m aquitard sequence in the northeast portion of the WB and used to determine *m*_v_ (*α*), *S*_S_, and *K*_h_ (site 1) (Smith et al. 2013, 2016, 2018). These VWPs have been monitoring pore pressure at site 1 for 16 years. However, with only a few in situ estimates of aquitard parameters reported in the literature, and limited use of VWPs to determine in situ* α* and *K*_*h*_ of aquitard formations, further work was required to determine the reliability of the method. To address this, an additional 34 VWPs were installed in five boreholes at a second site in the WB (site 2). The resulting dataset was used to evaluate the reproducibility of the methods through both glacial till and similar shale to augment the results obtained from site 1.

The results of this study confirm the long-term viability of fully grouted VWPs for monitoring pore pressure in low-*K* formations and represents one of the longest continuous in situ datasets for thick aquitards reported in the literature. The findings demonstrate that grouted-in VWPs can yield reliable and consistent estimates of key hydraulic properties, including *α*, *S*_S_, and *K*_h_ through the application of the *γ* method and recovery modeling in both glacial till and shale formations. The *α*, *S*_S_, and *K*_h_ results obtained from the VWPs installed at site 2 were consistent with the results from site 1 and verified that distinct depth dependent trends in hydraulic behavior over long- and short-terms are identifiable in the profile. The data presented in this study further supports using grouted-in pressure transducers to improve upon our understanding of complex low-*K* argillaceous deposits.

These findings contribute to the growing body of evidence supporting in situ methods for aquitard characterization and provide a high-resolution dataset that enhances the parameterization of regional groundwater flow models. This work has also identified several areas of future work that can be investigated in the long-term pore pressure record, including anomalous slip/strain behavior over short time periods, the effect of different drilling methods on pore pressure recovery and connectivity of instruments to adjacent formations, as well as the influence of low-*K* formations on regional groundwater flow in deep sedimentary basins.

## Data Availability

Datasets used in this research are available in Smith et al. (2013), Smith et al. (2016), and Smith et al. (2018). The raw pore pressure and barometric pressure data archived and analyzed for the study are available from the corresponding author upon reasonable request.
